# Supplementary material for: Cardiac muscle thin filament structures reveal calcium regulatory mechanism
Source: Nat Commun. 2020 Jan 9;11:153. doi: 10.1038/s41467-019-14008-1 (PMC6952405; doi:10.1038/s41467-019-14008-1)
Supplement: Supplementary file 1 — Supplementary Information [file 41467_2019_14008_MOESM1_ESM.pdf]

Supplementary Information

Cardiac muscle thin filament structures reveal calcium regulatory mechanism

Yamada et al.

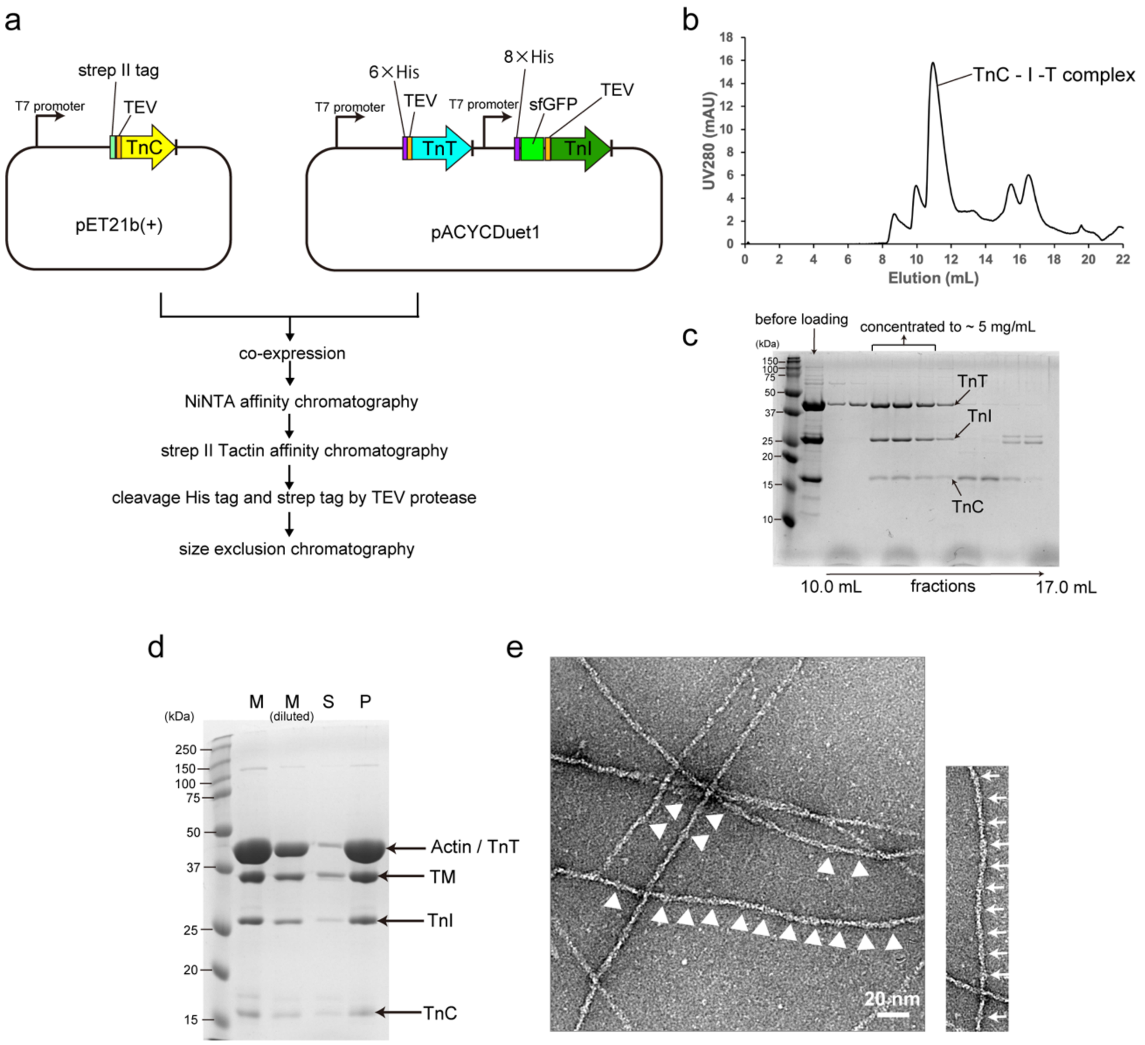

Supplementary Figure 1 Design of plasmids and expression and purification procedures. (a) Design of plasmids, (b) size exclusion column chromatography, (c) SDS-PAGE of the Tn ternary complex fraction, (d) SDS-PAGE of the reconstituted thin filament, M: mixture, S: supernatant, P: precipitate (e) negative stain EM image of the reconstituted thin filament with arrowheads indicating the positions of Tn cores.

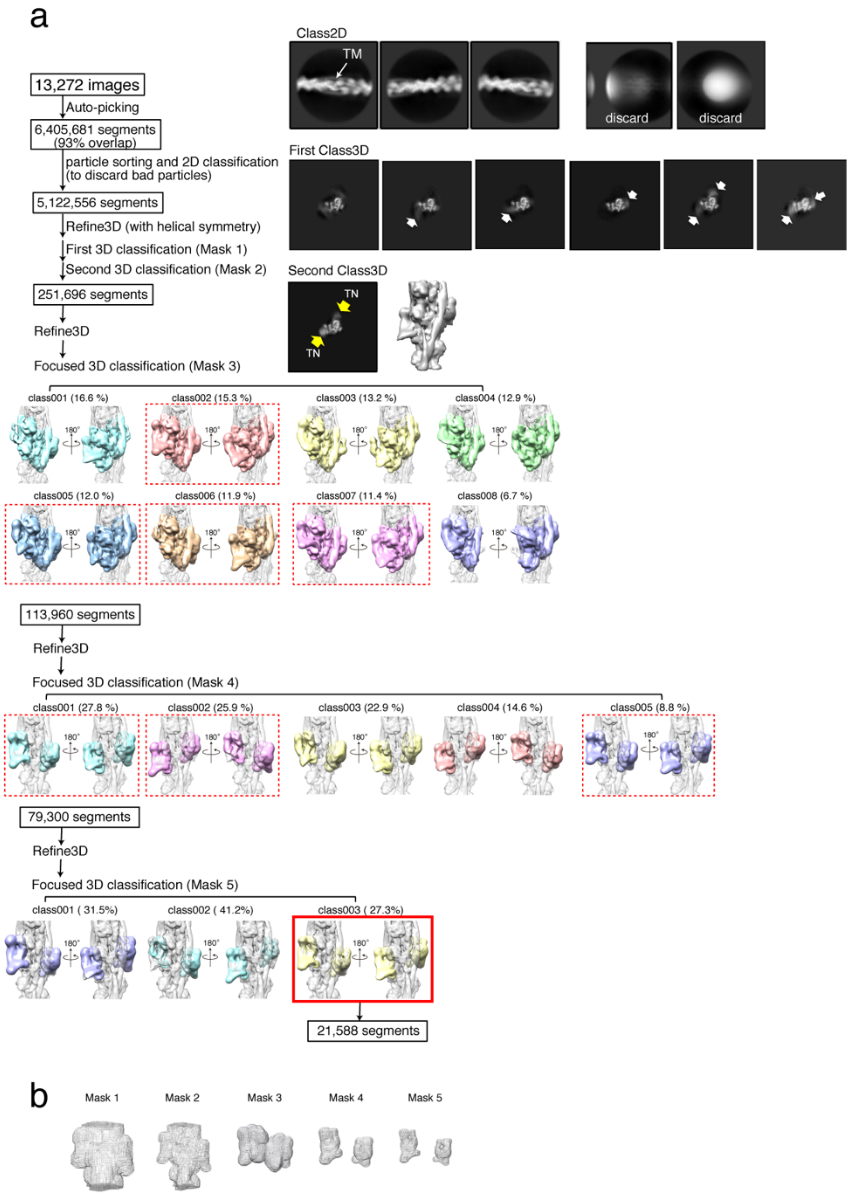

Supplementary Figure 2 Step by step description of the image processing sequence. (a) Image processing sequence by RELION 3.0. The good classes selected in each process is indicated by red square with either dotted or solid line, which is the one used for the final refinement. (b) Different masks used in the image processing sequence, with numbers in parentheses corresponding to those in the sequence in (a).

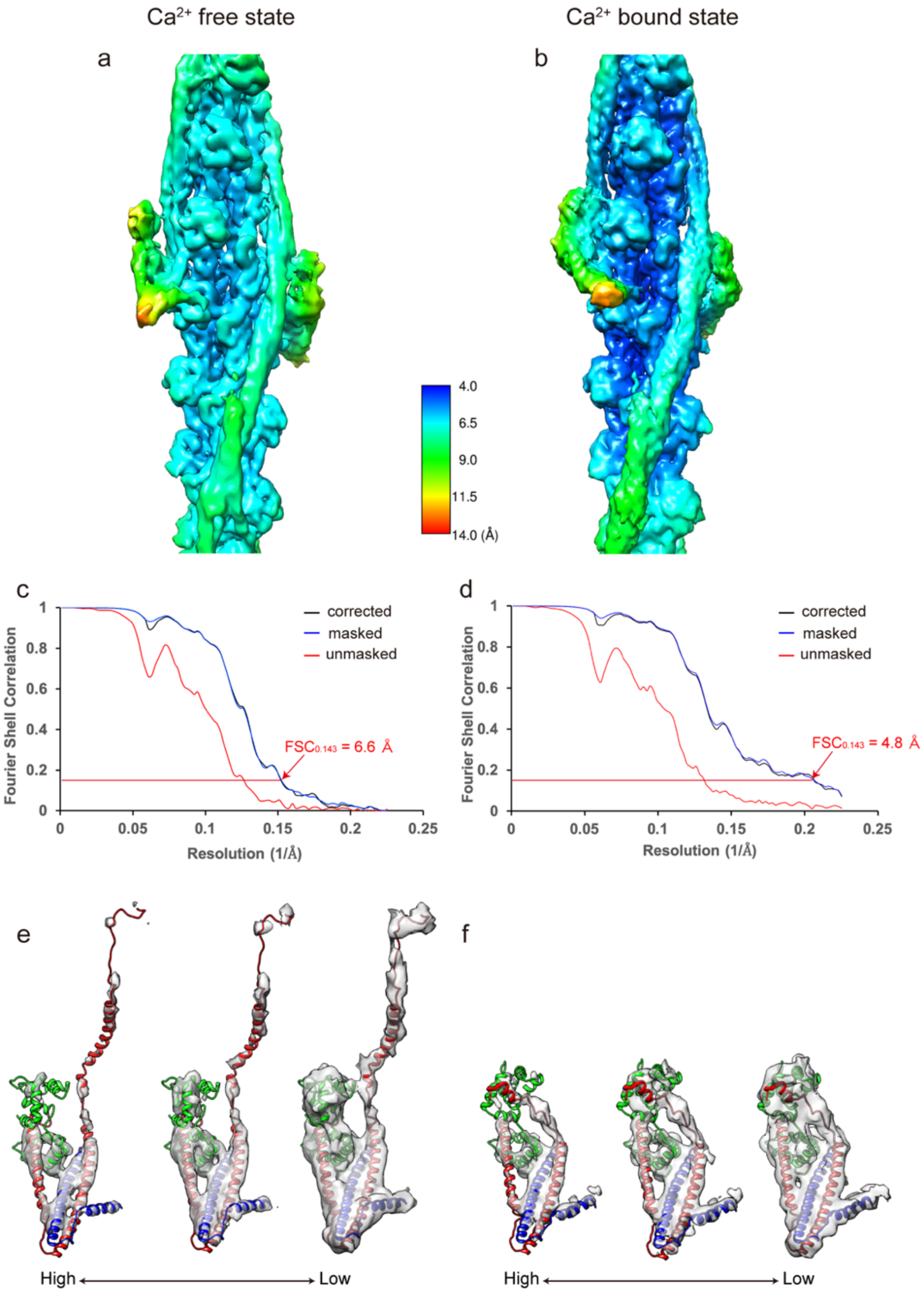

Supplementary Figure 3 Resolution and quality of the maps and fitted models. (a), (b) local resolutions of the reconstructed 3D volumes in the two states. (c), (d) Fourier shell correlation (FSC) curves indicating the overall resolutions of the 3D maps at an FSC of 0.143. (e), (f) 3D maps and fitted models of the Tn core, TnC_N and TnI_C with three different contour levels for the map. (e) Ca^{2+} free state, and (f) Ca^{2+} bound state.

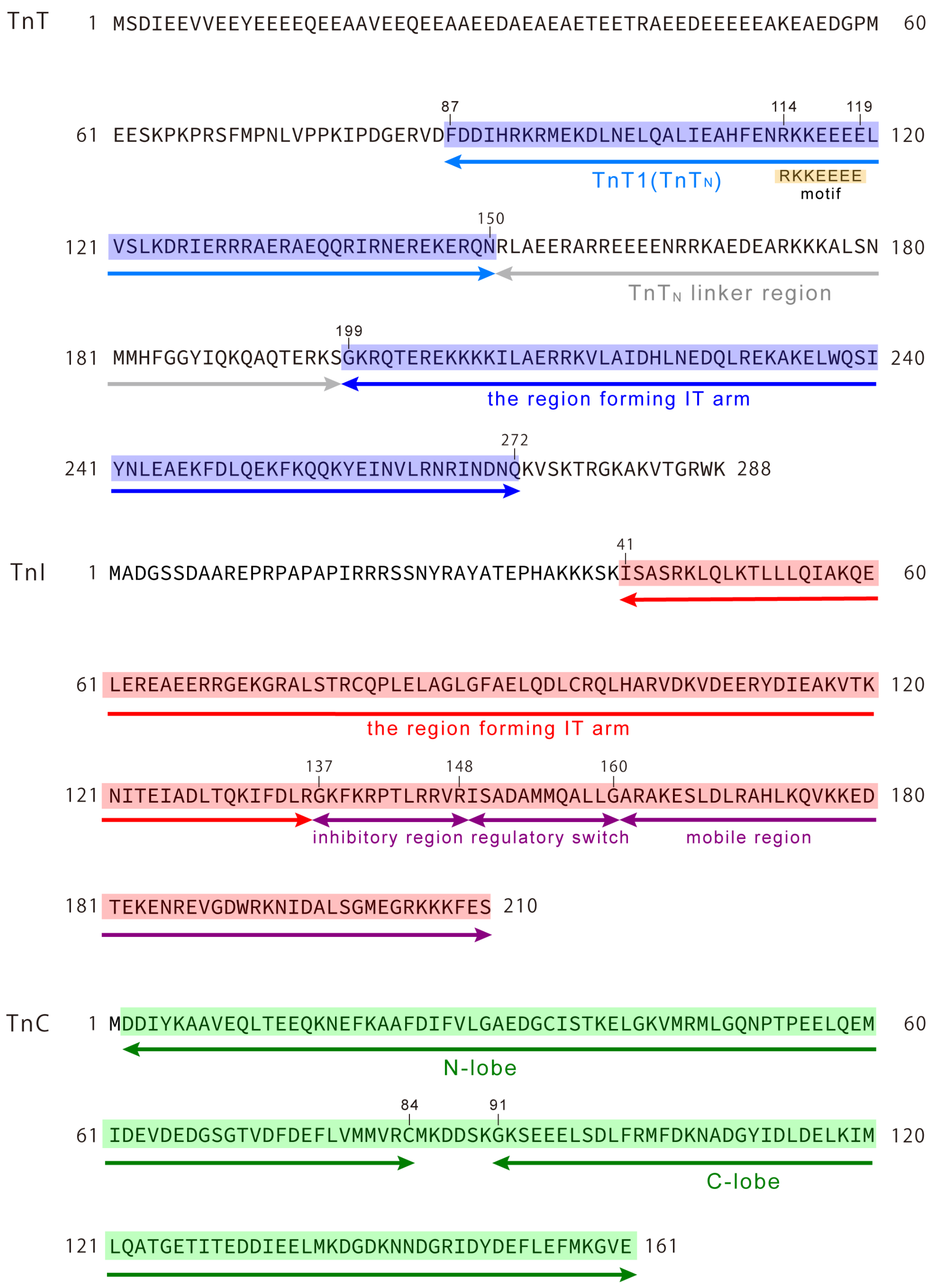

Supplementary Figure 4 Amino acid sequence of human cardiac TnT, TnI and TnC. The sequence regions of TnT, TnI and TnC covered in our model are colored purple, red and green, respectively.

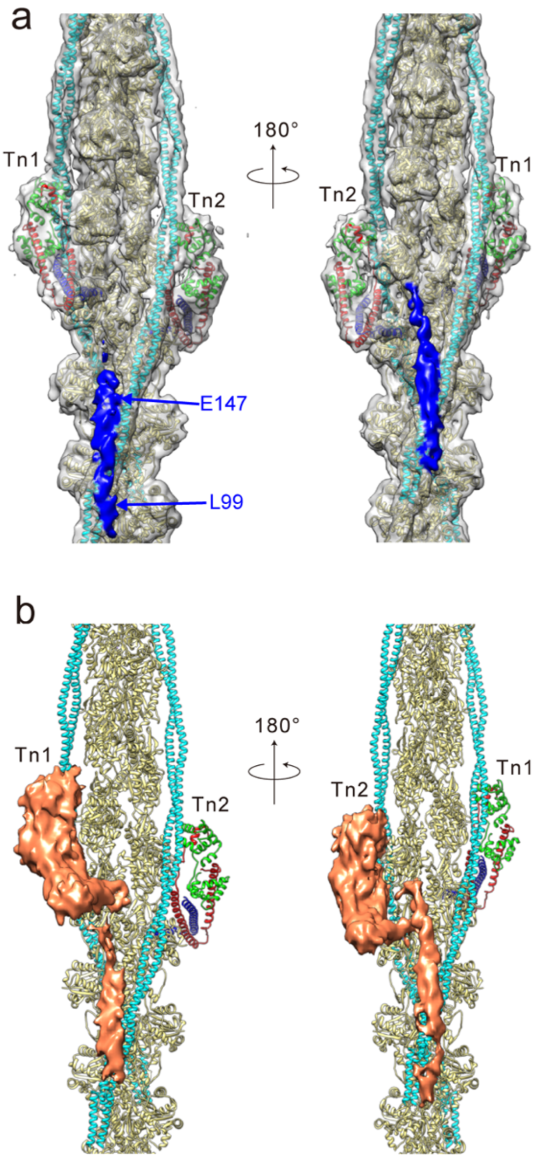

Supplementary Figure 5 The entire structures of the Tn ternary complexes in the Ca^{2+} bound state showing two different conformations of TnT_N. This figure is presented in the same scheme used for Fig. 2. (a) The entire structure of the Tn ternary complex is shown for each of the Tn pair, labeled Tn1 and Tn2, in front and back of the thin filament. The difference maps for TnT_N are colored dark blue below the Tn core. The difference in the relative positions of the Tn core and TnT_N between the pair is clear. The difference maps for TnI_C present above the Tn core in the Ca^{2+} free state are gone upon Ca^{2+} binding. (b) The difference map for the entire Tn ternary complex is contoured at a lower level to show the continuous volume of Tn in orange, for each of the Tn pair, again in front and back of the thin filament.

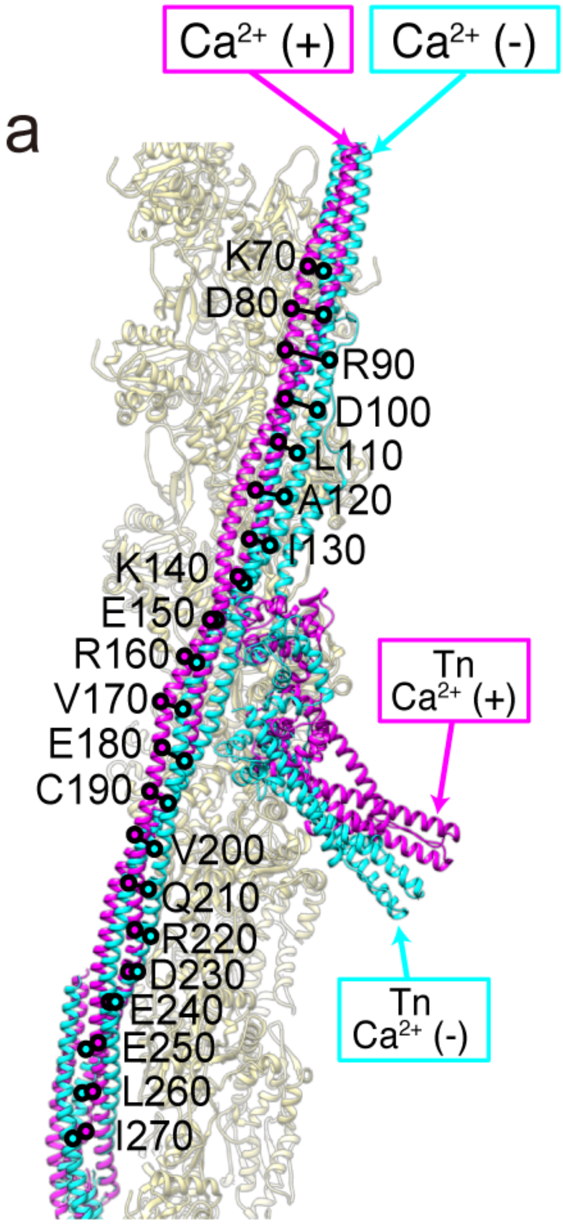

b

residue number	Cα-Cα distance (Å)
K70	4.9
D80	9.9
R90	13.5
D100	12.4
L110	7.6
A120	7.6
I130	6.7
K140	2.9
E150	3.2
R160	6.8
V170	9.6
E180	8.0
C190	6.8
V200	7.9
Q210	7.6
R220	4.7
D230	0.5
E240	3.2
E250	6.1
L260	2.1
I270	6.4

Supplementary Figure 6 Variation in the shift of Tm coiled coil on the surface of actin filament upon Ca^{2+} binding with measured shift distances. (a) The same figure as Fig. 4. (b) Measured distances between corresponding Cα atoms labeled by open circles. Tm and Tn core are colored blue and magenta for the Ca^{2+} free and bound states, respectively.

Supplementary Table 1 Summary of cryoEM data collection and image analysis

Data collection		
	Ca^{2+} free state	Ca^{2+} bound state
Microscope	CRYO ARM 200 prototype	
Camera	K2 (counted mode)	
Data acquisition	Auto / JADAS	
Pixel size (Å)	1.11 (counted mode)<br>2.22 (for reconstruction)	1.11(counted mode)<br>2.22 (for reconstruction)
Total electron dose ($e^-/Å^2$)	65	65
Number of frames	52	52
Frame time (sec)	0.3	0.3
Particle statistics		
Number of all micrographs	13,272	2,880
Number of picked segments	6,405,681 (93% overlap)	1,527,182 (93% overlap)
Number of segments (initial)	251,696	86,343
Number of segments (final)	21,588	23,374
Resolution (Å)	6.6	4.8

Supplementary Table 2 Primer sequences used in this study

Primer name	Type	sequence
AS_hcTM	Fwd	5' TTTAAGAAGGAGATATACATATGGCGAGCATGGATGCGATTAAAAAGAAAATGC
	Rev	5' CGACGGAGCTCGAATTCGGATCCTTAGATCGACGTCATGTCGTTC
StrepII_TEV	Fwd	5' GATATACATATGTGGAGCCACCCGCAGTTCGAAAAGGAAAACC
	Rev	5' GAATTCGGATCCGCCCTGAAAATACAGGTTTTCCTTTTCGAACTGCG
hcTNNC1	Fwd	5' ACCTGTATTTTCAGGGCATGGATGACATTTATAAAGCGGCC
	Rev	5' CGACGGAGCTCGAATTCGGATCCTTATTCAACACCTTTCATGAATTCCAG
His6_TEV_hcTNNT2	Fwd	5' CATCACCATCATCACCACGAAAACCTGTATTTTCAGGGCAGCGATATTGAAGAAGTGGTTG
	Rev	5' GCGCCGAGCTCGAATTCGGATCCTTATTTCCAACGACCC
His8_sfGFP	Fwd	5' GTATAAGAAGGAGATATACATATGCATCACCATCACCATCATCACCAC
	Rev	5' TCAGAGCTGCCATCCGCGCCCTGAAAATACAGGTTTTCTTTATACAGC
hcTNNI3	Fwd	5' TATAAGAAGGAGATATACATATGGCGGATGGCAGC
	Rev	5' TATCCAATTGAGATCTGCTTAAGATTCGAATTTTTTCTTACGACCTTCC

AS_hcTM: N-terminal Alanine-Serine extension human cardiac tropomyosin. hcTNNC1: human cardiac TnC. TnC was cloned into pET21b(+) with an N-terminal Strep-tag II and a TEV protease cleavage site using SLiCE method. His6_TEV_hcTNNT2: N-terminal His6-tag and TEV protease cleavage site were attached to human cardiac TnT. hcTNNI3: human cardiac TnI. Using SLiCE method, His8-tag and sfGFP and TEV protease cleavage site were fused to TnI at the N-terminus.
