# Supplementary material for: Cardiac muscle thin filament structures reveal calcium regulatory mechanism
Source: Nat Commun. 2020 Jan 9;11:153. doi: 10.1038/s41467-019-14008-1 (PMC6952405; doi:10.1038/s41467-019-14008-1)
Supplement: Supplementary file 3 — Description of Additional Supplementary Files [file 41467_2019_14008_MOESM3_ESM.pdf]

Description of Additional Supplementary Files

File Name: Supplementary Movie 1

Description: Conformational changes of the thin filament between Ca^{2+} free and bound states. Conformational changes of human cardiac thin filament between Ca^{2+} free and bound states, showing the rolling motions of the tropomyosin coiled coil on the surface of actin filament caused by the conformational changes of troponin upon Ca^{2+} binding to and release from troponin C.
